# Black Soldier Fly Larvae Meal *vs*. Soy Protein Concentrate Meal: A Comparative Digestibility Study in Barramundi (*Lates calcarifer*)

**DOI:** 10.1155/2024/3237898

**Published:** 2024-03-23

**Authors:** Richard Le Boucher, Weiqiang Chung, Jessalin Ng Kai Lin, Lydia Shun En Tan, Co Sin Lee

**Affiliations:** Precision Aquaculture Department, 1 Research Link National University of Singapore, Temasek Life Sciences Laboratory, Singapore 117604, Singapore

## Abstract

Black soldier fly larvae meal (BSFM) from *Hermetia illucens* has emerged as a dependable protein source in aquaculture. This study aimed to assess BSFM's digestibility in barramundi juveniles and compare it to soy protein concentrate meal (SPCM). Four diets (control, 30% BSFM; 30% SPCM; and commercial feed control) were tested on 1,800 barramundi juveniles (weight: 71.1 g) over 51 days in a recirculating aquaculture system (RAS). The final body weight (FBW) of fish fed with BSFM reached 222.2 (± 8.7), with a thermal-unit growth coefficient (TGC) of 4.33 (± 0.15) and a feed conversion ratio (FCR) of 1.04 (± 0.01). While BSFM and SPCM inclusion did not significantly impact FBW, body weight gain (BWG), TGC, or survival rates (*P*  > 0.05), FCR increased. BSFM significantly raised total feed intake (*P* < 0.05) but did not affect daily feed intake (*P*  > 0.05). Importantly, BSFM and SPCM inclusion did not alter diet apparent digestibility coefficient (ADC) for any nutrient groups (*P*  > 0.05), with BSFM showing high ADC for dry matter (76.8%), crude protein (93.2%), and gross energy (83.9%). No significant difference (*P*  > 0.05) was observed in these ADCs between BSFM and SPCM. The high digestibility of BSFM in warm seawater RAS (29.4°C) under high stocking density (33.7 kg m^−3^) supports its efficacy in contemporary barramundi farming.

## 1. Introduction

The global demand for aquatic food is on the rise globally, with projections indicating the need for an additional 26 million tons by 2030 [1]. This surge in demand is expected to be primarily met through aquaculture [2, 3]. Nevertheless, recent years have seen significant fluctuations in the supply, pricing, and quality of feed ingredients [4]. In response to this volatility, it has become imperative to identify and comprehensively characterize alternative ingredients [5]. These alternatives play a crucial role in providing formulators with the flexibility and adaptability needed to navigate the ever-changing landscape of aquatic feed production [6].

Among these alternative ingredients, insects are emerging as promising novel protein sources [7], as they have the potential to partially or completely replace commonly used fish and soybean meals [8]. Moreover, incorporating insects into aquafeed has the potential to reduce the environmental footprint of food production [9]. However, realizing this potential requires a comprehensive, case-specific assessment of environmental impact, safety, and economic factors [10]. Insects offer several advantages over other conventional feed resources. These include rapid growth, ease of reproduction, low feed conversion ratios (FCRs), and minimal requirements for arable land and water, depending on their life stage and feeding substrate [11].

In this evolving landscape, the black soldier fly (*Hermetia illucens*) has emerged as a highly efficient converter of a wide range of organic materials derived from food byproducts [12, 13]. While the availability of black soldier fly larvae meal (BSFM) for animal feed is on the rise, its nutritional value, particularly the crude lipid (CL) content, remains variable and relies on the type of substrate used as insect feed [12, 14]. Despite the increasing use of BSFM in aquafeed formulation, there is still a lack of comprehensive information regarding its amino acid and fatty acid content, digestibility [8], and its impact on the physical characteristics of extruded aquafeeds [15].

Only a limited number of studies have examined the impact of BSFM on barramundi (Asian seabass; *Lates calcarifer*) performance in small fish (1.7–6.7 g) [16–19], and none have estimated its apparent digestibility coefficients (ADCs) or digestible content. To date, the ADCs of BSFM have been estimated only in rainbow trout [20], turbot [21], and gilthead seabream [22]. Some studies have reported no significant impact of 10%–45% BSFM inclusion on diet apparent digestibility in gilthead seabream [22], rainbow trout [23], Atlantic salmon [24], and meagre [25]. However, adverse effects on digestibility were observed with BSFM inclusion of 40% in rainbow trout [15], 33% in turbot [21], 8.5% in yellow catfish [26], and, in some cases, above 10% in meagre [25].

For barramundi, a diadromous fish with a high trophic level (3.8 ± 0.60) [27] and a substantial protein requirement ranging from 40% to 65% [28], there is an increasing demand to identify sustainable alternative protein sources. This is particularly critical to support the growth of barramundi farming, especially given its popularity in Asia. The present study aimed to assess the in-vivo digestibility of BSFM and soy protein concentrate meal (SPCM) in 71.1 g barramundi.

## 2. Materials and Methods

### 2.1. Fish and System

One batch of 1,800 barramundi, with an average weight of 48.2 g, was transported from a commercial hatchery to the Marine Aquaculture Centre of the Singapore Food Agency. Prior to their transfer, the weight of 100 fish was accurately measured with a precision of 0.01 g. The *truncnorm* package [29], available in R software [30], was used to analyze the variance in fish weight after sorting. To ensure minimal size heterogeneity, fish were selectively chosen based on their individual body weight, ensuring a post-transfer coefficient of variation of 12%. After light sedation (using AQUI-S™ at 10 ppm), each fish was individually weighed (± 0.1 g), randomly distributed into 12 tanks, with each tank containing 150 fish and fed for a 15-day acclimatization period with a commercial feed designed for barramundi (Lucky Star EP6, Taiwan). The fish were then individually weighed, fed with experimental diets for 51 days, and weighed on the final day of the trial. The study utilized circular tanks with a water volume of 1,000 l, supplied with seawater maintained at 30.3 ± 0.5°C, in a recirculated aquaculture system (RAS). Before being transferred to the tanks at a flow rate of 1,500 l hr^−1^ (equivalent to 150% tank renewal per hour), the recirculated seawater underwent several treatment processes. These processes included passing through a drum filter, a moving bed biofilter, ultra-violet treatment (75 mJ cm^−2^), and ozonation (1.2–2.0 l min^−1^). Pure oxygen was accumulated with an Oxyport pressure swing adsorption system (Oxywise, Slovakia) and injected into the water through a 482 l Speece cone. Salinity, pH, nitrate, nitrite, total ammonia, and alkalinity in the biofilter were monitored daily. Dissolved oxygen levels (measured in mg l^−1^) and temperature were assessed in each tank every 10 min using standard electrochemical probes (OxyGuard, Denmark). A natural 12 : 12 hr photoperiod was maintained.

### 2.2. Experimental Diets

Three experimental diets and one commercial feed designed for barramundi (Lucky Star EP6, Taiwan) were tested. BSF and SPC experimental diets (Table 1) were the control diet, in which 30% of defatted BSFM and SPCM (Table 2) were respectively incorporated. The experimental diets were formulated using Wittaya software [31] to meet the specific nutritional requirements for barramundi of the desired size, including total proteins, digestible energy, and lipids (Table 3). These diets were manufactured at the R&D feed mill of the Marine Aquaculture Centre (Singapore). The diet formulation process began by sieving the ingredients to achieve a particle size of 500 *μ*m using a vibratory sifter. Subsequently, in 50 kg batches, the ingredients were thoroughly dry-mixed with a powder mixer (KSE-PM100, Kong Shiang Engineering, Singapore). The resulting diet premix was then conveyed to a 25 l twin-shaft preconditioner (25L V1, Clextral, Firminy, France) through a twin-screw feeder (DDSR20, Brabender, Germany) operating at 150 rpm. From there, the premix was processed through a twin-screw extruder (Evolum 25, Clextral, Firminy, France), equipped with intermeshing, corotating screws measuring 25 mm in diameter and 600 mm in length. The rotation speed of the extrusion screw was set at either 800 or 900 rpm. The screw configuration comprised a series of 24 feed screws (FS) and counter-threaded feed screws (CS), arranged in the following sequence from the barrel entrance: 4FS1, 10FS2, 2CS, 4FS2, and 4FS3, leading to the die. During the extrusion process, fish oil, heated to a temperature within the range of 50–50.5°C, was injected at the entrance of the barrel. The extruder barrel, consisting of six sections, maintained precise temperature control in each section using a series of heater collars. Throughout the extrusion process, die and barrel pressures were continuously monitored. Subsequently, the resulting pellets underwent a drying process at 60°C for a duration of 180–300 min, employing a boiling dryer (GFG-60, Mecflou, Singapore). In this study, moisture was introduced exclusively as liquid water, with no steam being utilized in this process. The total moisture incorporation rate ranged from 29.0% to 33.6% across all diets. Following extrusion and pellet drying, three samples from each diet were subjected to proximate composition analysis, and various pellet physical characteristics were recorded (Table 3). The pellet bulk density (BD) was determined by weighing loosely filled 1 l beakers (*n* = 3), and the pellet diameter (PellD, in mm) was calculated as an average from the measurement of 10 pellets per feed.

### 2.3. Feeding and Feces Collection

Over a period of 51 days, feed distribution to the fish was carried out manually by operators, providing feeds to satiation twice a day (from 9 : 30 AM to 11 : 30 AM and from 2 : 30 PM to 4 : 30 PM), 6 days a week. Feeding ceased once satiation was indicated by a decrease in feed intake, and it was confirmed when the first pellets reached the bottom of the tank. After a 30 min interval, any uneaten pellets were siphoned and quantified. The quantity of uneaten pellets was deducted from the initially distributed diets based on the individual pellet weights [32]. This adjusted value was then used to calculate the tank feed intake (TI) for each tank. In accordance with the recommendation of Blyth et al. [33], a 7-day acclimatization period was provided for barramundi to adjust to the diets before the first fecal collection. In each tank, feces were collected in the conical-shaped bottom tanks and upwelled into a swirl separator, which was cleaned and flushed daily. The collected fecal samples were subjected to centrifugation at 3,000 relative centrifugal force (rcf) for 15 min, with subsequent removal of the supernatant, elimination of scales, and freezing of the resulting pellets at −20°C. These frozen pellets were then subjected to oven-drying at 60°C for 24 hr, followed by chemical content analysis.

### 2.4. Chemical Analysis

Chemical analyses of the feed and feces were conducted by an accredited analytical service laboratory (Eurofins, Singapore), in accordance with the methods established by the Association of Official Analytical Chemists [34]. The calculation of Dry Matter (DM) was carried out through gravimetric analysis (AOAC 925.09) involving oven-drying at 100°C for 5 hr, followed by cooling in a desiccator. Crude lipid (CL) content was estimated through acid hydrolysis (AOAC 922.06). The determination of ash content (Ash) was performed gravimetrically, involving the measurement of the loss of mass after sample combustion at 550°C (AOAC 923.03). Total nitrogen (N) was estimated following pyrolysis and combustion (AOAC 968.06), and the level of crude protein (CP) was calculated as N × 6.25. Crude fiber (Fiber) was assessed after sample digestion with sulfuric acid and sodium hydroxide (AOAC 962.09). Nitrogen-free extract (NFE) was calculated using the DM-CP-CL-Fiber-Ash formula, and gross energy (GE) was determined using the formula 23.6^*∗*^CP + 39 ^*∗*^CL + 17.6^*∗*^(Fiber+NFE), as detailed in the National Research Council guidelines [35]. After digestion in hydrofluoric acid at 80°C, Yttrium was measured using inductively coupled plasma mass spectrometry (ICP-MS; Latech, Singapore).

### 2.5. Data Collection

The mean values of the initial and final fish body weights (FBW, in g) were employed to compute both the body weight gain (BWG, in g) and the thermal-unit growth coefficient (TGC) for each tank. TGC served as a standardized measure of growth [36], assumed to be unaffected by variations in body weight, time intervals, and water temperature differences [37, 38]. In this analysis, the TGC was calculated with a base of 20 to enhance its stability, especially in high-temperature conditions [39]:

(1) $$\begin{array}{c} {\text{TGC}}_{a}=1000\times \frac{{\text{FBW}}_{a}^{\left(\frac{1}{3}\right)}-{\text{IBW}}_{a}^{\left(\frac{1}{3}\right)}}{\sum_{i=0}^{51}\left(T_{i}-20\right)}, \end{array}$$

where FBW_*a*_ and IBW_*a*_ are the final and initial average FBW (g) in tank *a* and T_*i*_ (°C) is the water temperature on day *i*. The FCR in tank *a* was calculated as follows:

(2) $$\begin{array}{c} {\text{FCR}}_{a}=\left(\sum_{i=1}^{51}{\text{TI}}_{ai}/\text{qty of }{\text{fish}}_{ai}\right)/\left({\text{FBW}}_{a}-{\text{IBW}}_{a}\right), \end{array}$$

where TI_*ai*_ is the tank feed intake (g day^−1^) in tank *a* on day *i*. Daily feed intake (DFI, in g 100 g of fish^−1^ day^−1^) in tank *a* on day *i* was estimated as follows:

(3) $$\begin{array}{c} {\text{DFI}}_{ai}=100\times \left({\text{TI}}_{ai}/{\text{qty of fish}}_{ai}/{\text{BW}}_{ai}\right), \end{array}$$

where TI_*ai*_ is the feed intake in tank *a* on day *i*, qty of fish_*ai*_ is the fish quantity in tank *a* on day *i*, and BW_*ai*_ is the average FBW in tank *a* on day *i*, estimated using TGC and accumulated temperature unit (ATU) on day *i*. The ADCs (%) of DM, CP, CL, GE, nutrients, or nutrient groups in the experimental diets were calculated as follows:

(4) $$\begin{array}{c} {\text{ADC}}_{\text{parameter}}=\left[1-\left(\frac{Y_{\text{Diet}}\times {\text{Parameter}}_{\text{feces}}}{Y_{\text{feces}}\times {\text{Parameter}}_{\text{diet}}}\right)\right]\times 100, \end{array}$$

where *Y*_diet_ and *Y*_feces_ are the Yttrium content of the diet and feces, respectively, and Parameterdiet and Parameterfeces are the content of the nutritional parameter of interest (DM, CP, CL, GE, nutrient, or nutrient group) in the diet and feces, respectively. The ADCs of BSFM and SPCM ingredients (ADC_*ingr*_) were calculated according to Bureau et al. [40]:

(5) $$\begin{array}{c} {\text{ADC}}_{ingr}={\text{ADC}}_{test}+\left(\frac{\left(1-s\right)\times D_{Ref}}{s\times D_{ingr}}\right)\times \left({\text{ADC}}_{test}-{\text{ADC}}_{ref}\right), \end{array}$$

where ADC_*test*_ is the apparent digestibility coefficient of the feed containing the ingredient, *s* is the level of ingredient incorporation, *D*_*ref*_ is the nutrient content in the reference diet (as is), *D*_*ingr*_ is the nutrient content in the ingredient (as is), and ADC_*ref*_ is the apparent digestibility coefficient of the reference feed. Digestible contents were calculated based on diet composition and diet ADC, following the recommendations of Glencross et al. [41, 42].

### 2.6. Statistics

To analyze fish performance, a linear model [30] was used, and the effects were tested with a one-way ANOVA, with statistical significance set at *P*  < 0.05. Post-hoc comparisons of means between treatment groups were conducted using Tukey's HSD test to identify differences among the feeds. Residuals were assessed for normality using the Shapiro–Wilk test and for homogeneity of variance using Levene's test. For the survival rate and ADCs, an arcsine-square-root transformation was applied to meet the assumptions of normality and homogeneity of variance. Changes in DFI over time were analyzed using a two-way repeated measures ANOVA with fixed effects of day and diet, along with the day x diet interaction. If a significant interaction was observed, pairwise *t*-tests were performed between diets for each day.

## 3. Results

### 3.1. Growth Performance

**Figure 1:**
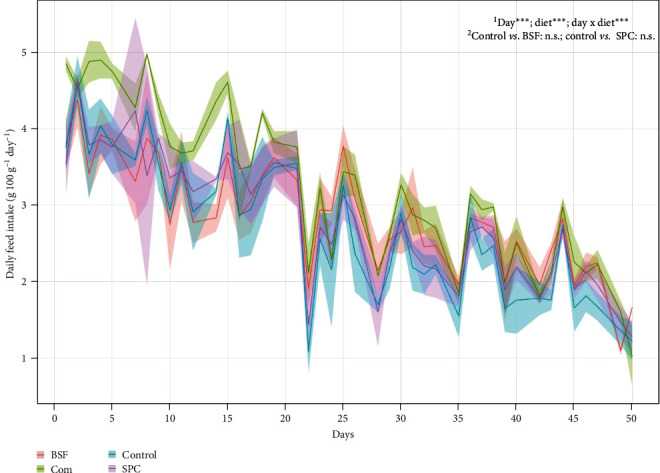
Changes in daily feed intake (g 100 g fish^−1^ day^−1^) in fish receiving control, black soldier fly (BSF), and soy protein concentrate (SPC) diets compared with those receiving commercial feed (Com). ^1^The day x diet interaction in the two-way repeated measures ANOVA was significant (*P*  < 0.001). ^2^Pairwise *t*-tests were computed every day to compare the diets.

Daily water temperature averaged 30.3°C ± 0.5, while dissolved oxygen levels was 8.83 ± 1.52 mg l^−1^. The survival rates exceeded 99.1% in all dietary treatments. The fish exhibited robust growth, with their body weight increasing by at least threefold over the 51-day growth period, leading to remarkable TGC values ranging from 4.16 to 4.33 (Table 4). The inclusion of 30% BSFM in the diet had no detrimental effects on fish survival rate, FBW, BWG, or TGC (*P*  > 0.05). Notably, the incorporation of BSFM had a positive impact on total feed intake (TFI; *P*=0.0125), albeit with a slightly higher FCR compared to the control diet (*P*  < 0.001). When compared to the commercial feed, the performance of the experimental diets was generally similar. Only FCR and TFI were lower in the experimental diets (*P*  < 0.05) compared to the chosen commercial barramundi feed. An analysis of feed intake trends over time revealed no significant differences in DFI between the BSF, SPC, and the control diets on any given day (*P*  > 0.8015). A power failure on day 21 caused a sudden decrease in oxygen supply, resulting in a similar reduction in DFI across all batches (Figure 1). The pellet BD of control diet and commercial feed were very similar, but differed between BSF (543.0 g l^−1^) and SPC (672.7 g l^−1^). Additionally, the PellD was slightly larger for commercial feed (8.1 mm) than control diet (6.6 mm).

### 3.2. Apparent Digestibility Coefficients

The diet's ADC estimates were notably high (Table 5) for DM (85.3%–89.0%), CP (93.3%–95.4%), CL (90.2%–94.0%), and GE (89.9%–92.3%). The inclusion of BSFM and SPCM did not have a significant effect on diet ADC for any nutrient groups (*P*  > 0.05). The ADC of BSFM DM was 76.8%, which was not significantly different from the digestibility of SPCM DM (86.0%). Additionally, the ADC of BSFM protein was notably high at 93.2%, with no significant difference compared to the apparent digestibility of protein in SPCM (99.9%). The digestive content for DM, CP, and GE in BSFM was determined to be 721.2 g kg^−1^, 483.2 g kg^−1^ DM, and 17.2 MJ kg^−1^ DM, respectively.

## 4. Discussion

### 4.1. BSFM Digestibility

This study marks the first attempt to assess insect meal digestibility in barramundi. The remarkably high protein ADC (93.2%) observed in this study competes with or even exceeds reported BSFM ADCs in other fish species, such as turbot (63.1%), seabream (84.4%), and rainbow trout (85%) [20–22]. In comparison to other ingredients [43], the BSFM used in this study exhibits high protein ADC values, closely resembling the protein ADC reported for barley or corn meals in barramundi [44]. Moreover, the BSFM employed in this research demonstrates a high-energy ADC, similar to values measured in barramundi for lupin kernel meals (*Lupinus angustifolius* and *Lupinus luteus*) [45]. These robust ADC findings offer valuable insights, particularly in light of previous concerns about barramundi's ability to digest chitin [19], which could have limited its use in aquafeed [46]. The significant difference observed between BSFM and SPCM in terms of lipid ADCs may be attributed to limitations in calculation methods, especially for ingredients with low lipid content, as seen in SPCM (Table 2). While these findings are encouraging for aquafeed formulators considering the incorporation of these materials into barramundi feed, it is important to acknowledge that the nutritional quality and chemical composition of BSFM can significantly vary depending on the insect feeding media [47], between products and batches [13], and potentially with different processing methods. Therefore, further trials using diverse sources of BSFM are crucial to gain comprehensive insights into its inclusion in barramundi diets. The field necessitates detailed data on insect processing, drying, substrate, and supply chain traceability to ensure the consistent utilization of BSFM in aquafeed.

### 4.2. Factors Affecting Insect Meal Digestibility

One potential explanation for the high BSFM digestibility observed in this study could be attributed to the use of extrusion for the experimental diet preparation. Prior studies examining BSFM digestibility in fish primarily relied on pressed pellets, produced using laboratory pellet mills or meat grinders, with only one study [36] utilizing extruded diets to estimate ADC. While extrusion is known to enhance the digestibility of plant protein [48], its impact on the digestibility of feed containing insect meal remains uncertain [49].

Another potential explanation for the high protein ADC for BSFM (93.2%) could be related to inaccuracies in estimating CP content during feed and waste chemical analysis. Indeed, the commonly used nitrogen-to-protein conversion factor (Kp) of 6.25 is known to vary among different food sources; for instance, fish meal and soybean flour have Kp values ranging from 5.4 to 5.7 and 5.4 to 5.6, respectively [50]. In the case of insect meal, determining an accurate Kp presents challenges due to uncertainties [51] and the variable presence of nondigestible protein in the insect cuticle [52]. In response to this challenge, Janssen et al. [53] proposed Kp values of 4.76 for *H. illucens* larvae and 5.62 for *H. illucens* extracts, taking into account nonprotein nitrogen and aiming to prevent protein content overestimation. Although ADC calculations are ratio-based and theoretically account for such overestimations, future studies could potentially improve accuracy by exploring the use of more precise Kp factors specifically designed for alternative protein sources like insect meals, thereby ensuring more accurate protein content calculations.

### 4.3. Impact of BSFM Inclusion on Barramundi Growth and Feed Intake

In this study, the inclusion of 30% BSFM in the diet did not significantly affect the growth of barramundi juveniles but did increase FCR. This percentage is higher than previous estimates conducted on barramundi juveniles (6–16 g) raised in freshwater, where the optimal inclusion rate of BSFM was approximately 15.4% [19]. Other research has primarily investigated the incorporation of BSFM as an additive in barramundi diets [16, 17], and one study highlighted the favorable impact of BSFM on bactericidal activity, expression of immune-related cytokines, and mucin cell production [18]. In other species, similar or lower inclusion rates of BSFM have been achieved while ensuring the absence of an impact on growth as the primary indicator. The reported maximum inclusion rates of BSFM were 14.7%–60% in salmonids [15, 24, 54–58], 17%–45% in marine fish [21, 22, 59–62], and 10.6%–22.3% in freshwater fish [63–65]. The rise in FCR noted in both BSF and SPC could potentially stem from the lower energy content of diets specifically formulated to optimize ingredient digestibility estimates.

The assessment of any new aquafeed ingredient is recommended to begin with an evaluation of its impact on palatability and the resulting variation in feed intake [6]. In some aquaculture species, the use of BSFM has been reported to reduce diet palatability [21, 55, 66, 67]. Conversely, in salmonids, incorporating 25%–30% BSFM into the diet has repeatedly been shown to have no impact on feed intake [55–58]. In this trial, there was a positive effect of high BSFM inclusion on TFI. Close monitoring of DFI also showed that there was not a single day when the inclusion of BSFM had a negative effect on DFI (*P*  < 0.05), not even during the first days of habituation to new diets. This initial phase of a feeding trial is usually considered to be the most critical, as it is during this time that the effects of a new diet on palatability are typically most accurately evaluated [6]. The acceptance of BSFM by barramundi suggests no palatability issues at the 30% inclusion level.

### 4.4. Optimizing Stocking Density for Digestibility Estimates

Nowadays, high fish stocking densities are common in intensive farming and can reach 40 kg m^−3^ in barramundi farms (pers.comm). However, high stocking density is known to negatively impact growth, feed conversion, and feed intake [68–70]. High stocking density can also induce stress in fish, leading to physiological and behavioral modifications that further reduce performance [71–73]. In barramundi, the maximum stocking densities, which do not compromise growth and FCR, have been previously estimated to be 15 kg m^−3^ in integrated RAS and 30 kg m^−3^ in brackish water cages [74, 75]. To avoid collecting digestibility estimates in conditions that would be too ideal compared to commercial farming, this study maintained a fish stocking density comparable to typical barramundi farming, reaching 33.7 ± 1.4 kg m^−3^ at the end of the trial.

Conversely, to avoid collecting digestibility estimates in suboptimal conditions compared to commercial farming, commercial feed was employed as a farm-representative control in this study. The absence of growth differences (*P*  > 0.05) between the commercial feed used and the 30% BSFM experimental diet confirms that the performance results of the experiment in seawater RAS align with current farm productivity expectations. Additionally, the barramundi in this study demonstrated a TGC of 4.2 ± 0.2, which is similar to or higher than values reported in previous research involving barramundi of similar sizes [76–81]. However, identifying commercial feeds that closely mirror the characteristics of experimental diets remains a persistent challenge. In this study, the marginally larger PellD of the commercial feed likely contributes to the higher TFI observed.

## 5. Conclusion

This assessment of BSFM digestibility in barramundi yielded favorable ADC values and evidenced no adverse effect on feed intake or performance. As BSFM is now among the insect species approved for use in aquaculture animal feed by the European Union [82], it is crucial to identify fish species that can efficiently utilize this new ingredient in the future. The performance results gathered in this study suggest that barramundi could be a promising candidate for incorporating more BSFM into aquafeed formulations.

## Figures and Tables

**Table 1:** Formulation (g kg^−1^) of control, black soldier fly (BSF), and soy protein concentrate meal (SPC) diets.

	Control	BSF	SPC
Fish meal, Skagen	500	350	350
BSFM^1^	—	300	—
SPCM^2^	—	—	300
Soybean meal	50	35	35
Wheat gluten meal	180	126	126
Wheat flour	161	113	113
Sardine oil	40	28	28
Tuna oil	40	28	28
Soy lecithin	10	7	7
Vitamin premix^3^	5	3	3
Mineral premix^3^	2	2	2
CaH_4_P_2_O_8_	10	7	7
Yttrium oxide	1	0.7	0.7

^1^Defatted black soldier fly (*H. illucens*) larvae meal (Inseact, Singapore). ^2^Soy protein concentrate meal (2 X-Soy 200, C.J. Selecta, Brazil). ^3^Vitamins and minerals were ANA Fish Vitamin Premix-199 and ANA Fish Mineral Premix-199 (Zagro, Singapore).

**Table 2:** Ingredients composition (in g kg^−1^ DM, unless otherwise specified).

	BSFM	SPCM
DM^1^	939.3	914.7
Ash	145.9	73.6
CP^2^	518.5	659.2
CL^3^	109.7	13.1
Fiber	91.1	40.6
NFE^4^	134.9	213.5
GE^5^	20.5	20.5

^1^Dry matter (g kg^−1^),^2^crude protein, ^3^crude lipid, ^4^nitrogen-free extract, and ^5^gross energy (MJ kg^−1^ DM).

**Table 3:** Composition (in g kg^−1^ DM, unless otherwise specified) and pellet characteristics of control, black soldier fly (BSF), and soy protein concentrate (SPC) diets compared with commercial feed (Com).

	Control	BSF	SPC	Com
*Proximate composition*				
DM^1^	922.0	901.6	903.6	913.6
Ash	93.2	109.5	84.2	120.4
CP^2^	598.8	577.9	616.4	545.1
CL^3^	158.4	156.4	121.7	159.8
Fiber	1.2	22.4	10.1	18.2
NFE^4^	148.4	133.9	167.6	156.5
GE^5^	22.9	22.5	22.4	22.2
Yttrium oxide	0.7	0.4	0.4	—
*Pellet physical characteristics*				
Diameter (mm)	6.6	6.5	6.1	8.1
Bulk density (g l^−1^)	594.0	543.0	672.7	593.0

^1^Dry matter (g kg^−1^), ^2^crude protein, ^3^crude lipid, ^4^nitrogen-free extract, and ^5^gross energy (MJ kg^−1^ DM).

**Table 4:** Mean values (*n* = 3) of survival, feed intake, growth, and feed conversion in juvenile barramundi receiving control, black soldier fly (BSF), and soy protein concentrate (SPC) diets compared with those receiving commercial feed (Com)^1^.

	Com	Control	BSF	SPC
Survival (%)	99.3 ± 0.7^a^	99.8 ± 0.4^a^	99.3 ± 1.1^a^	99.8 ± 0.4^a^
FBW^2^ (g)	236.5 ± 5.8^a^	217.3 ± 6.6^b^	222.2 ± 8.7^ab^	214.8 ± 2.8^b^
BWG^3^ (g)	165.0 ± 4.8^a^	146.7 ± 6.4^b^	151.1 ± 8.2^ab^	143.3 ± 2.9^b^
TFI^4^ (g)	178.6 ± 3.5^a^	141.3 ± 5.2^c^	156.9 ± 5.9^b^	148.5 ± 2.9^bc^
TGC^5^	4.60 ± 0.07^a^	4.25 ± 0.13^b^	4.33 ± 0.15^ab^	4.16 ± 0.07^b^
FCR^6^	1.08 ± 0.01^a^	0.96 ± 0.01^c^	1.04 ± 0.01^b^	1.04 ± 0.01^b^

^1^Different superscripts indicate significant differences among the values based on Tukey's HSD test (*P* ≤ 0.05); the values are presented as means ± SD. ^2^FBW, final body weight. ^3^BWG, body weight gain. ^4^TFI, total feed intake. ^5^TGC, thermal-unit growth coefficient in base 20. ^6^FCR, feed conversion ratio.

**Table 5:** Mean values (*n* = 3) of apparent digestibility coefficients (ADC) for control, black soldier fly (BSF), and soy protein concentrate (SPC) diets, mean values (*n* = 3) of ingredient ADC for black soldier fly larvae meal (BSFM) and soy protein concentrate meal (SPCM), and the digestible content of ingredients in barramundi (*L. calcarifer*)^1^.

	Control	BSF	SPC	SEM^2^
*Diet ADC (%)*				
DM	89.0^a^	85.3^a^	88.1^a^	2.63
CP	93.4^a^	93.3^a^	95.4^a^	2.15
CL	91.6^a^	90.2^a^	94.0^a^	2.79
GE	92.3^a^	89.9^a^	92.1^a^	2.24

*Ingredient ADC (%)*		BSFM	SPCM	
DM	—	76.8^a^	86.0^a^	4.79
CP	—	93.2^a^	99.9^a^	2.19
CL	—	85.8^a^	100.0^b^	3.64
GE	—	83.9^a^	91.4^a^	4.20

*Digestible content^3^*		BSFM	SPCM	
DM (g kg^−1^)	—	721.2	786.9	4.38
CP	—	483.2	658.3	1.41
CL	—	94.0	13.1	0.40
GE (MJ kg^−1^ DM)	—	17.2	18.8	0.86

^1^An arcsine-square-root transformation was applied to the ADC data; significant differences (*P*  < 0.05) among means are denoted by distinct superscript letters; in cases where apparent digestibility coefficients were greater than 100%, an absolute digestibility of 100% was assumed for practical reasons. ^2^Pooled standard error. ^3^Digestible content (in g kg^−1^ DM, unless otherwise specified) was determined through calculations involving ingredient composition (as detailed in Table 3) and ingredient ADC.

## Data Availability

Data available on request.
